# Effects of Leisure-Time Physical Activity on Cognitive Reserve Biomarkers and Leisure Motivation in the Pre-Diabetes Elderly

**DOI:** 10.3390/healthcare10040737

**Published:** 2022-04-15

**Authors:** Bo-Ram Kim, Seung-Taek Lim

**Affiliations:** 1 Department of Physical Education, College of Education, Korea University, Seoul 02841, Korea; brh@naver.com; 2 Olympic Studies Center, Kangwon National University, Chuncheon-si 24341, Korea

**Keywords:** prediabetes, elderly, physical activity, leisure-time, cognitive, biomarkers

## Abstract

The purpose of this study was to investigate the change in cognitive reserve biomarkers of the pre-diabetic individual according to the types of leisure-time physical activity (aerobic or resistance physical activity). The research subjects (*n* = 184) who participated in the survey were pre-diabetic and diabetic patients who were visiting university hospitals and welfare centers. The intervention subjects (*n* = 36) who were elderly females with pre-diabetes volunteered to participate in the study by performing regular physical exercise (aerobic or resistance exercise). The study participants were 65 years of age or older with pre-diabetes defined by a glycated hemoglobin (HbA1c) level of (5.7–6.4)%. All research subjects performed motivation and stress questionnaire survey. All intervention subjects participated in leisure-time physical activity (LTPA) for 12 weeks. Body composition, HbA1c, and cognitive reserve biomarkers were measured at baseline, and at 6 and 12 weeks. LTPA motivation confirmed that the LTPA participants had a high level of motivation. Stress confirmed that the stress level of LTPA participants was low. Two-way within-factor ANOVA revealed significant group × time interaction for weight (*p* < 0.05), BMI (*p* < 0.01), % fat (*p* < 0.001), SBP (*p* < 0.05), HbA1c (*p* < 0.001), BDNF (*p* < 0.001), and Beta-Amyloid 1–42 (*p* < 0.001). In both physical activity groups, HbA1c (*p* < 0.001), NGF (*p* < 0.05), BDNF (*p* < 0.05), and Cathepsin B (*p* < 0.05) improved significantly at 12 weeks, compared to baseline and 6 weeks. In the resistance physical activity group, Beta-Amyloid 1–42 (*p* < 0.01) and Homocysteine (*p* < 0.05) significantly decreased at 12 weeks, compared to baseline and at 6 weeks. The LTPA showed high levels of integrated and identified regulation among leisure motive types, and the level of stress was found to be low. The LTPA is effective in reducing the HbA1c levels of the pre-diabetes elderly. In addition, the pre-diabetes elderly were found to have increased NGF, BDNF, and cathepsin B, and decreased Beta-Amyloid 1–42 and homocysteine. Regular leisure-time physical activity has a positive effect on cognitive reserve biomarkers through improving glycemic control by reducing weight and % fat in the pre-diabetes elderly.

## 1. Introduction

Dementia caused by diabetes is a neurological disease that is emerging worldwide [1]. Several studies reported that diabetes (type 2 diabetes mellitus) was indirectly associated with greater cognitive decline through association with lower cortical thickness at baseline [2]; increased the precursors of amyloid deposition in the brain and pancreas [3]; and damaged the downstream of the insulin signaling pathway in the brain, which leads to hyperphosphorylation of the tau protein in the brain [4], which are hallmarks of dementia neuropathology.

Since diabetes is a risk factor for dementia and is very common in the elderly at risk of developing dementia, there is considerable potential for prevention, and interest in any intervention that can prevent or delay the onset of dementia is very high [5]. Pre-diabetes is a specific clinical condition of cognitive impairment, and physical exercise could promote the benefits of insulin regulation and sensitivity [6]. In addition, progression to clinically diagnosable dementia progresses in mild cognitive impairment (pre-dementia) by about 30% over about 3 years [7]. For this reason, the management and prevention of pre-diabetes and pre-dementia are very important.

Moreover, the mental stress of pre-diabetic or diabetic patients has a negative effect compared to the normal population. The scores of anxiety and depression in participants with pre-diabetes were significantly higher than those of participants with normal blood sugar levels [8]. Compared to the general population, pre-diabetic patients had 7.17 times higher mild depressive symptoms (95% CI (4.00–12.88)) and 7.77 times higher high depressive symptoms (95% CI (4.33–13.93)) [9]. Additionally, depression in diabetic patients was associated with a significantly higher risk of developing dementia than in patients with diabetes alone [10]. Physical inactivity can make diabetes worse, so motivation is needed to participate in physical activities. This motivation can improve physical activity management [11]. Physical activity is a very powerful factor in motivation for diabetics because it is most effective for weight reduction and glycemic control [12].

Regulatory exercise is receiving a lot of attention as a non-drug treatment for diabetes and dementia. Rowan et al. reported that a 12-week aerobic exercise program might improve glycemic control, visceral adiposity, and aerobic fitness in persons with pre-diabetes [13]. An aerobic training program conferred benefits in improving 2 h postprandial plasma glucose and HbA1c compared with resistance training for pre-diabetes [14]. In addition, a 12-week multicomponent exercise program (aerobic, resistance, and balance exercise) improved attention and dual-task ability in older women with mild cognitive impairment [15]. As such, research on disease improvement through exercise is actively in progress.

However, in this study, the most important preventive effect will be investigating the change in cognitive reserve biomarkers of the pre-diabetic individual according to the type of physical activity (aerobic or resistance exercise). These cognitive reserve biomarkers are the most representative Beta-Amyloid, brain neural plasticity factors (nerve growth factor (NGF), brain-derived neurotrophic factor (BDNF), and cathepsin-B), and homocysteine. These biomarkers are used to diagnose patients with mild cognitive impairment or dementia [16,17].

Therefore, diabetes has a great impact on dementia, and the importance of the prevention of pre-diabetes or at the pre-dementia stage is increasing. However, studies on which types of physical activity (aerobic vs. resistance) are effective in preventing cognitive function in pre-diabetic subjects are insufficient. In addition, it is very important to find out LTPA motivation and stress level. Thus, the purpose of this study was to investigate changes in cognitive reserve biomarkers according to leisure-time physical activity types (aerobic and resistant physical activity) in pre-diabetic patients and to identify LTPA motivational types and stress levels.

## 2. Methods

### 2.1. Participation

The research participants who participated in the survey were pre-diabetic and diabetic patients who were visiting university hospitals and welfare centers. The convenience sampling method was used as the sampling method, and after distributing a total of 270 copies, 184 subjects who responded that they participated in physical activity during leisure time were used for the final analysis.

The intervention participants, who were thirty-six elderly females with pre-diabetes, volunteered to participate in the study by performing regular physical exercise (aerobic or resistance exercise). The study participants were 65 years of age or older, and pre-diabetes was defined by a glycated hemoglobin (HbA1c) level of (5–6.4)% [18]. The participants were randomly assigned into the aerobic exercise group (*n* = 15), resistance exercise group (*n* = 12), and control group (*n* = 9).

The study subjects did not participate in a regular structured aerobic and resistance exercise program for at least four months prior to this study. The study goals and objectives, as well as the methodology, were explained to all subjects who agreed to participate to ensure complete understanding. This study complied with the ethical standards of the Declaration of Helsinki. Subjects signed an informed consent form before participation. This study was approved by the Kangwon National University Review Board for Human Subjects (KWNUIRB-2018-7-09-002-002).

The characteristics of the participants for intervention are shown in Table 1.

### 2.2. Measurement of Body Composition

The physical and anthropometric variables of both groups were measured. Body mass and height were measured to the nearest 0.1 kg and 0.1 cm, respectively, using a body composition analyzer (Inbody 720, Body Composition Analyzer; Biospace, Seoul, Korea). Body mass index (BMI) was calculated as weight in kilograms divided by height in meters squared.

### 2.3. Hematological Analysis

Fasting venous blood samples were collected from all participants at baseline, 6 weeks, and 12 weeks. Fasting was maintained for 12 h. Blood samples were collected on the following day, ensuring adequate sleep and refraining from radical movement as much as possible. All samples were drawn at 08:30 a.m. from antecubital veins. The samples were immediately centrifuged at 3500× *g* and 4 °C for 10 min, and the serum was stored at −80 °C until further analysis. Serum levels of cognitive biomarkers (nerve growth factor, brain-derived neurotrophic factor, cathepsin-B, homocysteine, and Beta-Amyloid 1–42) were measured using DueSet™ enzyme-linked immunosorbent assay (ELISA) kits (R&D systems, Minneapolis, MN, USA) according to the manufacturer’s instructions, as previously described.

### 2.4. Leisure-Time Physical Activity

The 12-week exercise program consisted of three days of supervised aerobic (Table 2a) and resistance (Table 2b) physical activity per week (i.e., Monday, Wednesday, and Friday) for each group.

Each exercise session included 10 min of warm up, 40 min for the main physical activity, and 10 min of cool down (i.e., 60 min in total). Aerobic physical activity consisted of rhythmic physical activity: vine step, manbo, twist, bumb, love repeat, love trick, and walking. Participants performed aerobic physical activity for 40 min at 55–65% of their heart rate reserve (HRR) and a 250−300 kcal burn of each main physical activity over 12 weeks. Resistance physical activity consisted of squat, lunge, chest press, vertical fly, lat pull down, long pull, and crunch for 40 min at 55−65% of their percent repetition maximum (%RM) and a 250−300 kcal burn of each main physical activity over 12 weeks.

The target heart rate and kcal burn were continuously monitored using a Polar M400 heart rate monitor (Polar Elector Co., Kempele, Finland).

A familiarization session was conducted during the first week. Participants were instructed to learn how to perform each resistance physical activity safely. Prior to participation, all subjects were informed of the risk associated with this study. Following informed consent, subjects completed questionnaires and pre-testing prior to participation in this study. They were familiarized with study procedures during the week prior to the implementation of aerobic and resistance physical activity.

### 2.5. Questionnaire Test

Leisure-time physical activity (LTPA) motivation is a perceived causal material scale (self-determination) developed by Vlachopoulos, Katartzi, Kontou, Moustaka, and Goudas [19], and validated by Song and Cheon [20]. The sub-factors of LTPA motivation were composed of 15 questions that included 4 factors: integrated regulation (e.g., I participate in LTPA because it is fun), identified regulation (e.g., I participate in LTPA because it is important to me), introjected regulation (e.g., I participate in LTPA because I feel obligated or pressured as a patient), and extrinsic regulation (e.g., I participate in the LTPA to get recognition from my doctor or family). The response type is composed of a 7-point Likert scale ranging from “not at all” (1 point) to “very much” (7 points), and a higher score means higher motivation. In addition, Cronbach’s alpha in this study ranged (0.905–0.918).

The stress scale was composed of the perceived stress inventory, a stress measurement tool of the National Health and Nutrition Examination Survey, used by Lee [21] for diabetic patients. The sub-factors of stress consisted of 20 questions, including 3 factors: burnout (e.g., I am mentally burnt out), depression (e.g., I have lost my confidence), anger (e.g., I am angry). The response type is composed of a 5-point Likert scale ranging from “not at all” (1 point) to “very much” (5 points), and a higher score means higher stress. In addition, Cronbach’s alpha in this study ranged (0.921–0.957).

### 2.6. Statistical Analysis

All results are reported as the mean ± standard deviation. All data were analyzed using SPSS version 25.0 (SPSS Inc., Chicago, IL, USA). First, the one-way ANOVA was used to assess group differences in baseline variables. For the three groups (aerobic, resistance, and control group) by three stages (baseline, 6 weeks, and 12 weeks), a two-way within-subject factor ANOVA was used to examine whether the exercise type and time influenced cognitive reserve biomarkers. A Bonferroni test was used for post hoc analysis. Second, Cronbach’s coefficient was calculated to measure the reliability of the questionnaire, and descriptive statistical analysis was performed to determine the leisure motivation and stress of LTPA participants. Statistical significance was accepted at a = 0.05.

## 3. Results

### 3.1. Demographic Characteristics for Pre-Diabetes-Elderly-Only Questionnaire

The Table 3 shown demographic characteristics for the pre-diabetes-elderly-only questionnaire.

This study analyzed the motivation for LTPA, stress, and health-related quality of life of the diabetic patients who participated in LTPA.

### 3.2. LTPA Motivation and Stress

The Table 4 shown descriptive statistical analysis on LTPA motivation and stress.

The motivation is distributed from the lowest 1 point to the highest 7 point, where the higher the score, the higher the motivation level. As a result of the analysis, both integrated regulation (M = 5.03) and identified regulation (M = 5.49), which are the sub-factors of LTPA motivation, were higher than the intermediate index (4 points), confirming that LTPA participants had a high level of motivation. On the other hand, introjected regulation and extrinsic regulation showed a low index.

Stress is distributed from the lowest 1 point to the highest 5 point, where the higher the score, the higher the stress level. As a result of the analysis, burnout (M = 1.90), depression (M = 1.69), and anger (M = 1.64), which are the sub-factors of stress, all showed lower indices than the intermediate index (3 points), confirming that the stress level of LTPA participants was low.

### 3.3. Change in Body Composition and Glycated Hemoglobin (HbA1c) Level after Intervention

The Table 5 shown Changes in body composition and HbA1c.

Two-way within-factor ANOVA revealed significant group × time interaction for weight (*p* < 0.05), BMI (*p* < 0.01), % fat (*p* < 0.001), SBP (*p* < 0.05), and HbA1c (*p* < 0.001). Post hoc analysis using the Bonferroni test indicated that in the aerobic physical activity group, weight, BMI, and SBP significantly decreased at 12 weeks from the values at baseline and 6 weeks. In addition, in the resistance physical activity group, % fat was significantly decreased at 12 weeks, compared to the baseline and 6-week values.

Post hoc analysis using the Bonferroni test indicated that in both physical activity groups, the levels of HbA1c were significantly decreased at 12 weeks compared with those at baseline and 6 weeks. However, in the control group, HbA1c levels were significantly increased at 12 weeks compared with those at baseline and 6 weeks.

### 3.4. Change in Cognitive Reserve Biomarkers after Intervention

**Figure 1:**
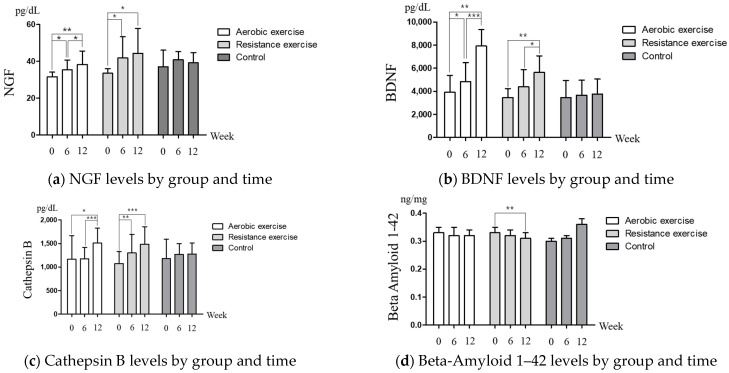

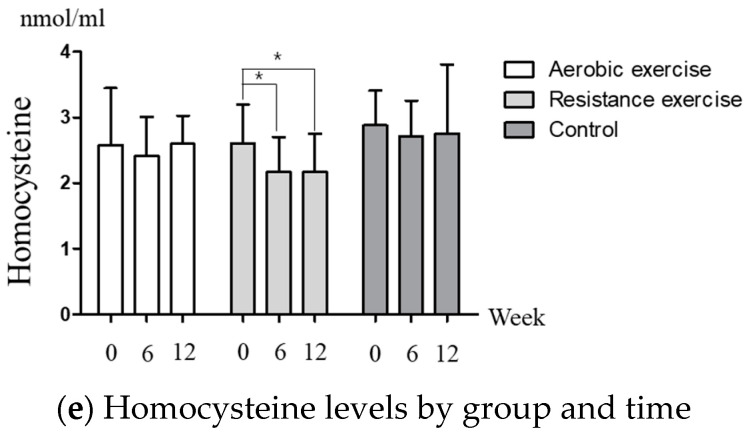
Cognitive reserve biomarker levels by group and time (**a**) NGF, group: 2.442 (0.102), time: 13.201 (<0.001), group × time: 1.224 (0.309). (**b**) BDNF, group: 2.614 (0.088), time: 19.595 (<0.001), group × time: 5.304 (0.001). (**c**) Cathepsin B, group: 0.080 (0.924), time: 11.981 (<0.001), group x time: 2.131 (0.087). (**d**) Beta-Amyloid 1–42, group: 0.291 (0.751), time: 3.837 (0.031), group × time: 8.475 (<0.001) (**e**) Homocysteine, group: 0.907 (0.422), time: 1.801 (0.181), group × time: 0.750 (0.565). * *p*-value was analyzed by post hoc (Bonferroni test), * *p* < 0.05, ** *p* < 0.01, *** *p* < 0.001.

Figure 1 shows the changes in levels of cognitive reserve biomarkers after intervention. Two-way within-factor ANOVA revealed significant group × time interaction for BDNF (*p* < 0.001) and Beta-Amyloid 1–42 (*p* < 0.001). Post hoc analysis using the Bonferroni test indicated that in both physical activity groups, NGF (Figure 1a), BDNF (Figure 1b), and Cathepsin B (Figure 1c) were significantly increased at 12 weeks from the values at baseline and 6 weeks. In addition, in the resistance physical activity group, Beta-Amyloid 1–42 (Figure 1d) and Homocysteine (Figure 1e) were significantly decreased at 12 weeks, compared to the baseline and 6-week values.

## 4. Discussion

This study investigated the change in cognitive reserve biomarkers of the pre-diabetic individual according to the type of leisure-time physical activity (aerobic or resistance physical activity). The main finding of this study was that after 12 weeks, in both physical activity groups, NGF, BDNF, and Cathepsin B of the cognitive reserve biomarkers were increased. In the resistance physical activity group, Homocysteine and Beta-Amyloid 1–42 of the cognitive reserve biomarkers were decreased. In addition, in both physical activity groups, HbA1c levels were significantly decreased after 12 weeks.

In the type of motivation for leisure-time physical activity, the higher the integrated and identified regulation, the more active the participation in physical activity that is induced and the enjoyment that is experienced [22]. According to the theory of self-determination, among the motivation types, integrated regulation and identified regulation are motivation types with a high level of self-determination, and subjects participate in physical activity because it corresponds with the personal value and importance of the activity and the values pursued by the individual [23]. In the case of diabetes patients, the higher the motivation for integrated regulation and identified regulation, the more they participate in regular physical activity [24]. In fact, patients with pre-diabetes who participate in regular physical activities showed high identified regulations among motivation types [25]. For regular physical activity in pre-diabetes and diabetic patients, it is important to motivate integrated and identified regulation with a high level of self-determination among motivation types [26].

Mild cognitive impairment and Alzheimer’s disease reduced cognitive function, which is accompanied by high stress and poor quality of life [27]. Since there is a correlation between diabetes and Alzheimer’s disease, proper management through regular physical activity for diabetic patients is an important way to prevent cognitive decline and Alzheimer’s disease [28]. As a result of this study, the reason why the stress is low is because of the usual participation in LTPA. Participation in LTPA has a beneficial effect on stress reduction [29]. Compared to those who do not actually participate in LTPA, the stress levels of LTPA participants tend to be significantly lower [30].

Type 2 diabetes is associated with dementia and cognitive decline, in which vascular damage and glucose-mediated process and other metabolic disorders can also play a role [31]. In addition, pre-diabetes may affect cognition through altered brain metabolism and may be more vulnerable to the negative effects of glucose intolerance [32]. This study conducted two types of physical activity in the elderly in the pre-diabetes and observed cognitive reserve biomarkers in relation to changes in brain metabolism. Representative cognitive reserve biomarkers include NGF [33], BDNF [34], Cathepsin B [35], and Beta-Amyloid 1–42 [36]. Although homocysteine is not directly associated with brain metabolism, the cognitive status of type 2 diabetes is associated with homocysteine levels, which is an independent risk factor for cognitive exacerbation and is associated with endothelial damage [37].

The NGF, BDNF, and Cathepsin B were increased after 12 weeks’ regular physical activity in women [38]. Physical exercise increased neuroplasticity via neurotrophic factors, such as BDNF and NGF, which improved cognitive function [39]. BDNF and Cathepsin B levels were inversely correlated with weekly hours of exercise [40]. In this study, the concentrations of NGF, BDNF, and Cathepsin B were increased in aerobic and resistance physical activity after 12 weeks. However, these concentrations did not change in the control group. Özbeyli et al. reported that all exercise types are mediated by an increase in hippocampus or cortical NGF immunoreactive neurons, whose increase may stimulate neuroplasticity [41]. Increased metabolic activity during exercise increases BDNF in both circulating platelets and vascular endothelial cells due to increased stress that the brain and/or radical endothermic cells contribute to circulating BDNF [42]. Cathepsin B might mediate the benefits of exercise for brain function through several pathways and shows increased hippocampal Cathepsin B gene expression [43]. Exercise or physical activity has a consistent effect on the volume of the hippocampus observed among the elderly, and the volumetric change of the hippocampus is associated with exercise [44]. Plasma concentration of Beta-Amyloid 1–42 was evaluated lower with higher levels of physical activity and exercise [45]. In this study, the concentration of Beta-Amyloid 1–42 also showed a significant decrease in resistance physical activity after 12 weeks. Although there was no significant difference in aerobic physical activity, there was a tendency to decrease, while in the control group, there was a tendency to increase. These results did not show according to the type of physical activity of aerobic or resistance exercise, but it is believed to have an effect on Apolipoprotein E (APOE) ε4 [46]. Indeed, the relationship between habitual levels of physical activity and brain Beta-Amyloid 1–42 only exists for carriers of the APOE ε4 allele, which may be associated with a higher level of physical activity mitigating the increased risk of Beta-Amyloid 1–42 deposition conferred by APOE ε4 transport [47]. Increased total plasma homocysteine is associated with an increased risk of cognitive impairment and dementia [48]. Deminice et al. reported that resistance training reduced plasma homocysteine concentration but aerobic training did not. In this study, the concentration of homocysteine also showed a significant decrease only in the resistance physical activity group after 12 weeks [49]. Previous studies reported that acute aerobic exercise induced increased homocysteine [50,51]. Homocysteine may damage brain tissue through multiple pathways, and it directs nitric oxide to vascular endothelial cells or causes dysfunction [52]. Perhaps due to this influence, the result of homocysteine appeared differently, depending on the type of aerobic or resistance physical activity.

The management of blood sugar levels in patients with pre-diabetes is very important in the preventive stage. Previous studies reported that physical activity and/or exercise greatly improve glycemic control and visceral adiposity. Malin et al. reported that aerobic training increased pancreatic function adjusted to skeletal muscles in relation to the improvement of glucose tolerance in adults with pre-diabetes [53]. Huang et al. reported that resistance training also showed beneficial effects on insulin resistance and glycemic control in pre-diabetes patients [54]. These results demonstrated that weight and fat mass reduction due to regular physical activity may improve through the activation of mitochondrial oxidation ability and reduction in endogenous glucose production as an underlying mechanism [54]. This study showed significant decrease in HbA1c after 12 weeks of aerobic and resistance physical activity. As a result of this study, aerobic and resistance physical activity would have contributed to weight and % fat reduction and had a positive effect on glycemic control.

The present study has some limitations. First, it is necessary to determine whether it contributes to the cognitive reserve biomarkers level of patients with cognitive decline or dementia. Although this study focuses on preventing the morbidity of cognitive decline and dementia in the pre-diabetic stage, it is necessary to analyze patients with cognitive decline or dementia. Second, we did not receive a questionnaire from participants who were involved in physical activity. This is due to the third limitation of the sample size being small, which limits our ability to determine the significance of the results. Therefore, additional studies with larger sample sizes are required.

## 5. Conclusions

The study confirmed that the pre-diabetic elderly who participated in LTPA had high levels of integrated and identified regulation among leisure motive types, and the level of stress was found to be low. In addition, the results of this study indicated that leisure-time physical activity is effective in reducing the HbA1c levels of the pre-diabetes elderly. In addition, the pre-diabetes elderly were found to have increased NGF, BDNF, and cathepsin B, and decreased Beta-Amyloid 1–42 and homocysteine. In regular aerobic and resistance physical activity, positive changes in lipolysis pathway factors in adipose tissue promote lipid degradation and reduce fat mass [55], and improved glycemic control had a positive effect on cognitive reserve biomarkers [56]. Thus, regular leisure-time physical activity has a positive effect on cognitive reserve biomarkers by improving glycemic control by reducing weight and % fat in the pre-diabetes elderly.

## Figures and Tables

**Table 1:** Characteristics of the participants for intervention.

Variable	Group	Mean ± SD	*p*-Value
Age (year)	Aerobic Exercise (*n* = 15)	70.47 ± 5.57	0.123
	Resistance Exercise (*n* = 12)	72.25 ± 5.07	
	Control (*n* = 9)	67.78 ± 2.33	
Height (cm)	Aerobic Exercise (*n* = 15)	153.7 ± 4.73	0.134
	Resistance Exercise (*n* = 12)	149.9 ± 6.30	
	Control (*n* = 9)	154.7 ± 6.50	
Weight (kg)	Aerobic Exercise (*n* = 15)	62.45 ± 10.34	0.355
	Resistance Exercise (*n* = 12)	57.32 ± 9.10	
	Control (*n* = 9)	59.52 ± 6.51	
BMI (kg/m^2^)	Aerobic Exercise (*n* = 15)	26.50 ± 4.48	0.506
	Resistance Exercise (*n* = 12)	25.37 ± 2.84	
	Control (*n* = 9)	24.86 ± 2.23	
Percent fat (%)	Aerobic Exercise (*n* = 15)	38.33 ± 7.34	0.711
	Resistance Exercise (*n* = 12)	38.19 ± 5.81	
	Control (*n* = 9)	36.32 ± 3.63	
SBP (mmHg)	Aerobic Exercise (*n* = 15)	141.0 ± 14.09	0.684
	Resistance Exercise (*n* = 12)	137.4 ± 9.92	
	Control (*n* = 9)	137.2 ± 10.28	
DBP (mmHg)	Aerobic Exercise (*n* = 15)	68.33 ± 6.88	0.303
	Resistance Exercise (*n* = 12)	72.17 ± 6.46	
	Control (*n* = 9)	70.78 ± 5.40	

Mean ± SD. BMI; body mass index, SBP; systolic blood pressure, DBP; diastolic blood pressure.

**Table 2:** (**a**) Aerobic physical activity. (**b**) Resistance physical activity.

(**a**)			
**Items** **Station**	**Exercise**	**Intensity**	**Time**
1	Warm up		10 min
2	Vine step	HRR 55∼65% 250∼300 kcal/day	40 min
3	Mambo		
4	Twist		
5	Bumb		
6	Love repeat		
7	Love trick		
8	Walking		
9	Cool down		10 min
(**b**)			
**Items** **Station**	**Exercise**	**Intensity**	**Time**
1	Warm up		10 min
2	Squat	%RM 55∼65% 250∼300 kcal/day	40 min
3	Lunge		
4	Chest press		
5	Vertical fly		
6	Lat pull down		
7	Long pull		
8	Crunch		
9	Cool down		10 min

**Table 3:** Demographic characteristics for the pre-diabetes-elderly-only questionnaire.

	Category	*n*	%	Variable	Category	*n*	%
Gender	Male	54	29.3	LTPA Frequency	Fewer than 3 times a week	101	54.9
	Female	130	70.7				
					3 or more times a week	83	45.1
Age	69.74 ± 10	184	100				
Other disease	None	66	35.9	LTPA Amount	Less than 1 h	134	72.8
	Hypertension	88	47.8				
					Over 1 h	50	27.2
	Etc.	30	16.3				
Total		184	100.0	Total		184	100.0

LTPA; Leisure-time physical activity.

**Table 4:** Descriptive statistical analysis on LTPA motivation and stress.

Variable	Category	Minimum	Maximum	M ± SD
LTPA motivation	Integrated regulation	1.00	7.00	5.03 ± 1.52
	Identified regulation	1.00	7.00	5.49 ± 1.32
	Introjected regulation	1.00	6.50	3.72 ± 0.93
	Extrinsic regulation	1.00	7.00	3.15 ± 1.09
Stress	Burnout	1.00	5.00	1.90 ± 0.94
	Depression	1.00	5.00	1.69 ± 0.91
	Anger	1.00	5.00	1.64 ± 0.92

LTPA; Leisure-time physical activity.

**Table 5:** Changes in body composition and HbA1c.

Variable		0 Week ^a^	6 Weeks ^b^	12 Weeks ^c^	Post hoc	*F*-Value (*p*-Value)
Weight (kg)	AE	62.45 ± 10.35	61.84 ± 10.29	59.75 ± 9.19	a > b > c	G: 0.935 (0.403) T: 19.254 (<0.001) G × T: 3.559 (0.011)
	RE	57.32 ± 9.10	57.12 ± 9.01	55.87 ± 7.14	-	
	CG	59.52 ± 6.51	58.58 ± 6.40	58.79 ± 6.61	a > b	
BMI (kg/m^2^)	AE	26.50 ± 4.48	26.27 ± 4.53	25.34 ± 3.96	a > b > c	G: 0.526 (0.596) T: 14.373 (<0.001) G × T: 3.774 (0.008)
	RE	25.37 ± 2.84	25.32 ± 2.83	24.87 ± 2.21	-	
	CG	24.86 ± 2.23	24.46 ± 2.25	24.57 ± 2.20	a > b	
Fat (%)	AE	38.33 ± 7.34	37.93 ± 8.10	36.60 ± 7.52	-	G: 0.199 (0.820) T: 34.508 (<0.001) G × T: 7.331 (<0.001)
	RE	38.19 *±* 5.81	37.53 ± 6.69	35.27 ± 5.32	a > b > c	
	CG	36.32 ± 3.63	35.67 ± 3.38	35.90 ± 2.72	a > b	
SBP (mmHg)	AE	141.0 ± 14.09	137.0 ± 12.72	133.27 ± 14.29	a > c	G: 0.476 (0.626) T: 1.018 (0.367) G × T: 2.776 (0.034)
	RE	137.7 ± 9.92	137.6 ± 14.77	143.0 ± 11.79	-	
	CG	137.2 ± 10.28	135.9 ± 8.11	131.8 ± 7.92	b > c	
DBP (mmHg)	AE	68.33 ± 6.88	68.67 ± 7.45	67.53 ± 7.34	-	G: 2.298 (0.116) T: 0.128 (0.880) G × T: 0.835 (0.508)
	RE	72.17 ± 6.46	73.67 ± 12.30	75.08 ± 8.53	-	
	CG	70.78 ± 5.40	70.44 ± 5.90	68.56 ± 6.31	-	
HbA1c (%)	AE	6.16 ± 0.29	6.09 ± 0.29	5.79 ± 0.40	a > b > c	G: 0.449 (0.642) T: 17.255 (<0.001) G × T: 21.441 (<0.001)
	RE	6.08 ± 0.39	6.05 ± 0.33	5.61 ± 0.39	a > b > c	
	CG	5.84 ± 0.31	5.81 ± 0.41	6.08 ± 0.28	a < b < c	

Mean ± SD, AE; aerobic exercise, RE; resistance exercise, CG; control group, BMI; body mass index, SBP; systolic blood pressure, DBP; diastolic blood pressure, HbA1c; glycated hemoglobin, G; Group, T; Time, G × T; Group × Time. ^a^: 0 week; ^b^: 6 weeks; ^c^: 12 weeks.

## Data Availability

Data available on request due to restrictions eg privacy or ethical. The data presented in this study are available on request from the corresponding author.
